# On the Virtues of Tinct. Colchici in Chorea

**Published:** 1816-10

**Authors:** Thomas Raven

**Affiliations:** House Pupil to the Norfolk and Norwich Hospital.


*For the London Medical and Physical Journal.*

*On the Virtues of Tinct. Colchici in Chorea;* by Mr. THOMAS RAVEN, House Pupil to the Norfolk and Norwich Hospital.

MILES STANLEY, æt. 17, of a spare habit of body and sickly complexion, presented himself at our hospital on the 27th of July last, and was admitted as an in-patient under the care of Dr. Alderson; his disorder was chorea sancti viti, under its most grotesque and fantastic forms, and had acted with great violence for a length of time. On the day he was admitted, he was ordered, without any preparatory purgative, to take thirty drops of tinct. colchici every four hours. In the course of two days, the symptoms were considerably abated, and at the end of a fortnight so completely removed, that he was allowed to walk into the city for two hours. He continued the medicine in rather an increased dose while he remained in the house; and, as there was no appearance of the disorder returning, he was discharged charged, cured, on September 7, 1816. If you should think this case worthy insertion, in my next (with your leave) I shall send you a still more extraordinary case of the spasmodic kind.

*Norwich; Sept.* 12, 1816.

